# A rare association of intussusception and celiac disease in a child: Associação rara entre intussuscepção e doença celíaca em criança

**DOI:** 10.1590/1516-3180.2016.0092220616

**Published:** 2016-09-19

**Authors:** Vanessa Pacini Inaba Fernandes, Elizete Aparecida Lomazi, Maria Angela Bellomo-Brandão

**Affiliations:** I MD, MSc. Attending Physician, Pediatric Gastroenterology, Universidade Estadual de Campinas (Unicamp), Campinas, SP, Brazil.; II MD, PhD. Professor of Pediatrics, Pediatric Gastroenterology, Universidade Estadual de Campinas (Unicamp), Campinas, SP, Brazil.

**Keywords:** Child, Intussusception, Celiac disease, Diarrhea, Gluten-free diet, Criança, Intussuscepção, Doença celíaca, Diarreia, Dieta livre de glúten

## Abstract

**CONTEXT:**

Intussusception is a common cause of acute intestinal obstruction in the pediatric population and it is normally idiopathic. Rare cases of chronic intussusception require investigation with greater attention.

**CASE REPORT:**

We present a clinical case of a three-year-old boy with aqueous diarrhea, abdominal distension, vomiting and weight loss over a two-month period. During the investigation, abdominal ultrasound showed imaging of intussusception. The intraoperative findings showed the intussusception had resolved spontaneously. In further investigation, it was found that the diarrhea was malabsorptive and, after the patient underwent upper gastrointestinal endoscopy, a diagnosis of celiac disease was made. After a gluten-free diet was introduced, the patient showed complete remission of symptoms and regained weight, and normal growth was reestablished.

**CONCLUSION:**

If the clinical presentation of intussusception is unusual, etiological investigation should be undertaken. In this case report, celiac disease was the underlying cause.

## RESUMO

**CONTEXTO:**

Intussuscepção é uma causa frequente de obstrução intestinal aguda em pacientes pediátricos, e geralmente é idiopática. Raros casos de intussuscepção crônica devem ser investigados com maior atenção.

**RELATO DE CASO:**

Apresentaremos um caso clínico de um menino de três anos com diarreia aquosa, distensão abdominal, vômito e perda de peso por dois meses, cuja investigação por ultrassonografia abdominal revelou imagem de intussuscepção. Os achados intraoperatórios mostraram que a intussuscepção havia se resolvido espontaneamente. Em investigação subsequente, foi identificado que a diarreia era malabsortiva e, após a realização de endoscopia digestiva alta, foi feito diagnóstico de doença celíaca. Foi iniciada dieta com restrição de glúten e o paciente teve remissão completa dos sintomas, recuperou peso e o crescimento foi restabelecido.

**CONCLUSÃO:**

Caso a apresentação clínica de intussuscepção não seja a habitual, deve-se prosseguir a investigação etiológica. No caso clínico apresentado, doença celíaca foi a causa subjacente.

## INTRODUCTION

Intussusception is a common cause of small bowel obstruction in children under five years of age, and its classical symptoms include acute abdominal pain, red currant jelly stools and abdominal mass. However, these classical symptoms are not always present and it sometimes mimics acute viral gastroenteritis, with diarrhea and vomiting. Invagination of the proximal bowel into the distal bowel results in venous congestion and bowel wall edema.^1^ If not promptly diagnosed and treated, this condition can lead to arterial obstruction, bowel necrosis and perforation. Almost 90% of the etiology of intussusception in children is ileocolic and idiopathic.^1^ The lead point of the invagination is lymphoid hyperplasia of the small bowel, but Meckel’s diverticulum, polyps and trauma can also lead to this problem. Intussusception is diagnosed by means of abdominal ultrasound and can be treated surgically or non-surgically.

This case report describes the presence of intussusception in a child with a chronic history of diarrhea, which is an unusual presentation in children that should indicate the possibility of a different diagnosis. After the case description, there is a brief discussion with a systematic search of data in the PubMed, Cochrane and LILACS databases (Table 1).

**Table 1.**
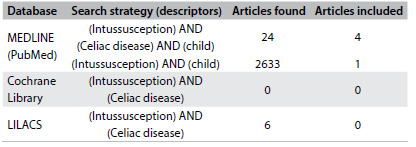
Database search results for the relationship between intussusception and celiac disease and child. Search performed on February 22, 2016

## CASE REPORT

A three-year-old boy with a history of liquid diarrhea for a couple of days, several times a day, with intermittent vomiting and without blood on stools or fever, was first seen by a primary care physician. At physical examination, the child was found to be hydrated, without abdominal pain or distention, and was able to take oral fluids and medication. He was diagnosed as having acute gastroenteritis and was advised to take oral hydrate solution and to return to the primary care center if the symptoms worsened. Fourteen days later, the child returned and the mother complained that he had been suffering from intermittent diarrhea, vomiting and abdominal distention. At examination, he did not show any weight loss, was hydrated and had an overall good clinical condition. At this point, the diagnosis was persistent diarrhea and he was advised to start a lactose-free diet, take albendazole for five days and collect parasitological stool samples.

There was no short-term follow-up on this case by the primary care team because the family requested a second opinion from a different general pediatrician. The second physician made the interpretation that the child possibly had lactose intolerance and ordered a lactose intolerance test with blood analysis. When the patient returned to the original primary care center, two months after the initial onset, he was still presenting aqueous diarrhea three to six times a day, abdominal distension, intermittent vomiting and now weight loss. Parasitological stool samples were negative and blood analysis suggested an iron deficient anemia. The lactose intolerance test was not available at that moment. The diagnosis at this point was chronic diarrhea with probable malabsorption. The child was referred to a tertiary gastroenterology center for further investigation, with upper gastrointestinal endoscopy, serum xylose tests and stool analysis for fat and pH.

**Figure 1.**
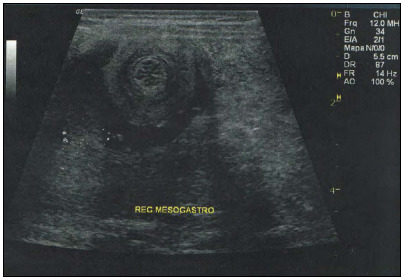
Abdominal ultrasound image showing an onionskin-like part of the small bowel suggestive of intussusception.

Abdominal ultrasound was performed and identified an onionskin image of part of the small bowel (Figure 1), suggestive of intussusception. On the day of the ultrasound, the patient presented a distended but soft palpable abdomen without abdominal pain and was referred for hospital admission. After hospital admission, laparoscopic intervention demonstrated dilation of the proximal small bowel, without any mass or enlarged lymph nodes.

The intraoperative findings showed that the intussusception had resolved spontaneously. During the hospital stay, further investigations on chronic diarrhea and malabsorption were conducted, and the most relevant results were the following: low albumin of 3.2 g/dl; increased international normalized ratio (INR) of 1.95, which resolved after vitamin K administration; low serum sodium (Na) of 133 mEq/l; high presence of fat in stools (12 g of fat in 161.7 g of stool after 72 hours) and normal stool pH of 6.0; low serum xylose of 6.08 mg/dl, suggestive of malabsorption; negative HIV serological test; normal immunoglobulin A (IgA) antibody level of 247 mg/dl; and normal sweat test (Na of 44.01 mEq/l and Cl of 35.6 mEq/l). All of these laboratory and reference values are shown in Table 2. To investigate malabsorption, upper gastrointestinal endoscopy was performed, and macroscopic evaluation showed a mosaic pattern of mucosa suggestive of celiac disease. On microscopic evaluation, inflammation of the duodenum mucosa was observed along with atrophic intestinal villi and crypt hypertrophy, compatible with Marsh III celiac disease, which was further confirmed by elevated tissue transglutaminase antibody concentration of 200 U/ml.

**Table 2.**
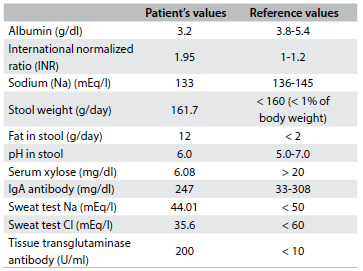
Patient’s laboratory result values and reference values for normality

IgA = immunoglobulin A.

The child was then administered a gluten-free diet and his weight, stool consistency and stool frequency recovered completely, thus resolving the abdominal distention. He was discharged after a month in hospital, with body weight of 16.1 kg. One month later, at an outpatient visit, he was weighing 17.4 kg. One year after admission, his weight had increased to 19.8 kg.

## DISCUSSION

Idiopathic intussusception is a common cause of small bowel obstruction in children between the ages of 3 months and 5 years and has been recently correlated with underlying celiac disease.^1^^,^^2^^,^^3^^,^^4^ It is the most common cause of intestinal obstruction at pediatric ages and needs prompt intervention, which may be surgical or nonsurgical.

Non-operative methods for treating intussusception include barium enema and hydrostatic or pneumatic reduction. While non-operative methods are used more commonly than surgical intervention, the latter may still be needed when there are complications (peritonitis, perforation or profound shock) or when non-operative intervention is unsuccessful, or in situations of lack of a trained professional for non-invasive approaches.^1^

Most cases of intestinal intussusception at pediatric ages are idiopathic, and the lead point is generally lymphoid hyperplasia of the small bowel. The classical symptoms of intussusception include acute abdominal pain, red currant jelly stools and abdominal mass in a child with normal nutritional status. If the child fails to thrive and has chronic diarrhea, pain or blood stools, further investigation should be conducted after treating the intussusception, as was done in our patient. The differential diagnoses include Meckel’s diverticulum, polyps, trauma, celiac disease and enteropathy-associated T-cell lymphoma, among others.^1^

Celiac disease is a chronic inflammatory intestinal disease that occurs in about 1 to 2% of the general population. Untreated celiac disease has therefore been correlated with intussusception, and this association has been documented by a number of case reports.^2^^,^^3^^,^^4^ The proposed cause of intussusception in cases of celiac disease is diffuse inflammation and thickening of the intestinal wall, which lead to hyperperistalsis and increased dilation of the small bowel. This, in turn, could be the lead point for intussusception, which can develop singly or multiply, chronically and with self-resolution or the need for surgical intervention. Less frequently, but with worse prognosis, this lead point could also be associated with a focal lead point in lymphomas.^4^

The majority of studies on this subject are case descriptions or series of cases, and there is a lack of case-control or randomized clinical studies. In a recent retrospective study on patients undergoing imaging for abdominal pain, intussusception was found more frequently in patients with untreated celiac disease (less than nine months before celiac disease was diagnosed) than in the general population (1.2% versus 0.07% respectively).^2^ In a large case-control study evaluating the risk of late celiac disease in patients with intussusception, no significant association was found.^3^ Nonetheless, using a prospective cohort approach, a post-hoc analysis found that 12 out of 29,060 individuals with celiac disease were given a diagnosis of intussusception after the onset of celiac disease, with a modest but significant increase risk of intussusception after celiac disease had been diagnosed (odds ratio, OR = 1.95; 95% confidence interval, CI = 1.01-3.77; P = 0.046). In that study, patients with celiac disease without symptoms were not serially investigated with abdominal imaging, which may have reduced the absolute numbers of patients with subclinical intussusception who did not seek acute care and were unaccounted for.

Because intussusception in celiac patients may be chronic and painless, it is probably underdiagnosed. Patients with celiac disease may undergo imaging for chronic abdominal pain and intussusception may be identified by chance, as it is usually not the first differential diagnosis in these cases. While in adults most cases of intussusception are investigated because it is always a pathological condition, in children this is only done when an abnormal clinical presentation or physical examination ensues, since it is considered to be a common cause of idiopathic intestinal obstruction.

What makes our case description original is the chronic presentation of intussusception with spontaneous resolution, without the need for surgical intervention. This finding has only infrequently been described. The majority of case descriptions found in the literature describe an emergency situation of intussusception, in a child who failed to thrive.^4^^,^^5^ It will only be determined whether spontaneous resolution in these situations is more frequent when routine abdominal ultrasound becomes part of the celiac disease protocol workup. A large serial study on pediatric populations with celiac disease with routine abdominal ultrasound might help in resolving this question.

## CONCLUSION

Intussusception is a common cause of idiopathic acute intestinal obstruction in children. If the clinical presentation is unusual, this may prompt further investigation. In this case, celiac disease was the pathological condition behind the findings, with clinical presentation of chronic painless intussusception, without any need for surgical intervention and with signs and symptoms of malabsorption.
